# Giant Cerebral Tuberculoma Masquerading as Malignant Brain Tumor – A Report of Two Cases

**DOI:** 10.7759/cureus.10546

**Published:** 2020-09-20

**Authors:** Prity Agrawal, Subash Phuyal, Rajesh Panth, Pratyush Shrestha, Ritesh Lamsal

**Affiliations:** 1 Radiology, Upendra Devkota Memorial National Institute of Neurological and Allied Sciences, Kathmandu, NPL; 2 Neuroimaging and Interventional Neuroradiology, Upendra Devkota Memorial National Institute of Neurological and Allied Sciences, Kathmandu, NPL; 3 Pathology, Upendra Devkota Memorial National Institute of Neurological and Allied Sciences, Kathmandu, NPL; 4 Neurosurgery, Upendra Devkota Memorial National Institute of Neurological and Allied Sciences, Kathmandu, NPL; 5 Anaesthesiology, Tribhuvan University Teaching Hospital, Institute of Medicine, Kathmandu, NPL

**Keywords:** brain tumor, magnetic resonance imaging, tuberculoma, tuberculosis

## Abstract

Giant cerebral tuberculoma is an uncommon but serious form of tuberculosis. We report two patients who had a single, large lesion on magnetic resonance imaging (MRI) of the brain. Both patients underwent neurosurgery for the excision of the mass lesion as neuroimaging findings were suggestive of a brain tumor. Tuberculoma was later diagnosed on histopathological examination. We want to highlight that cerebral tuberculomas can mimic malignant brain tumors, as the clinical, laboratory, and radiologic features of cerebral tuberculomas are nonspecific.

## Introduction

Tuberculosis of the central nervous system is less common compared with other organ systems [1]. Central nervous system involvement in the form of meningitis, encephalopathy, tuberculous arteriopathy, tuberculoma, abscess, infarct, or miliary parenchymal lesions is seen in only 2-5% patients with tuberculosis [2]. Cerebral tuberculoma is the least common presentation of central nervous system (CNS) tuberculosis [3]. A single, large tuberculoma is often confused with a brain tumor in patients who do not have overt clinical features of tuberculosis infection. Fever and other constitutional symptoms are often absent in patients with cerebral tuberculomas. We present two patients who had a giant cerebral tuberculoma that mimicked a malignant brain tumor on magnetic resonance imaging (MRI). Both the patients underwent craniotomy and excision of the intracranial mass, which was later diagnosed as tuberculoma on histopathological examination.

## Case presentation

Case 1

**Figure 1:**
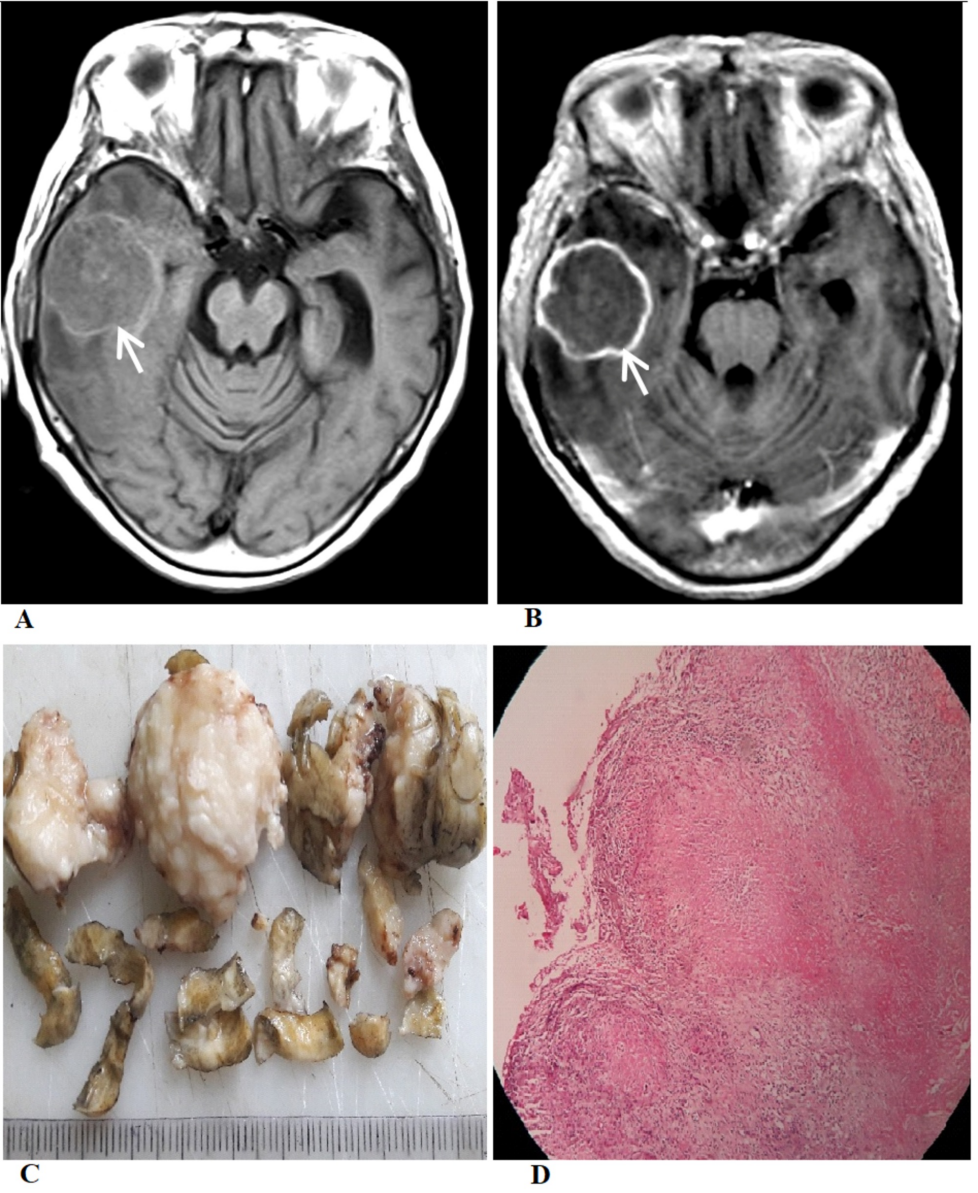
Radiologic and pathologic images (A) Axial T1-weighted MRI image shows a relatively well-defined iso-intense lesion with a hyperintense rim involving the right temporal lobe (white arrow). (B) Post-contrast T1-weighted MRI image shows a smooth ring enhancement (white arrow). (C) The gross specimen shows multiple irregular pieces of grayish-brown tissue. (D) Microscopic examination shows caseous necrosis with epithelioid cell granulomas bordered by lymphocytes.

A 73-year-old woman was referred to the hospital with an intermittent low-grade fever for one month. She also had three episodes of loss of consciousness and two episodes of generalized seizures. There was no history of nausea, vomiting, blurring of vision, or headache. She was taking antipyretics but was not on other medications. On examination, the Glasgow Coma Scale (GCS) score was 15. There were no signs of meningism or focal neurological deficits. In laboratory tests, the results of the erythrocyte sedimentation rate (ESR), C-reactive protein (CRP), and other routine biochemical tests were normal. The chest X-ray was unremarkable. Brain MRI revealed a peripheral enhancing solitary space-occupying lesion measuring 4 x 3.9 x 3.7 cm^3^ in the right temporal lobe, with significant perilesional vasogenic edema (Figure 1).

MR spectroscopy showed an increase in choline peak with a decrease in the N-acetylaspartate peak. The choline/N-acetylaspartate and choline/creatine ratio were high, suggestive of high-grade glioma. Considering her age, the initial radiological differential diagnoses included cystic glioblastoma and intracranial metastasis. The work-up for metastasis, including contrast-enhanced computed tomography scans of the chest and abdomen, was non-contributory. Because of the clinical presentation, the mass effect caused by the lesion, and uncertain clinical diagnosis, the patient underwent a right frontotemporoparietal craniotomy for the excision of the mass on the third day of admission. The mass was firm, grayish, avascular, and partly adherent to the dura with well-demarcated tissue planes. After complete excision, the specimen was sent for histopathological and microbiological examinations. The histopathological examination found a necrotizing granulomatous inflammatory lesion, consistent with the diagnosis of tuberculoma. Anti-tuberculosis treatment (ATT) with isoniazid, rifampicin, pyrazinamide, and ethambutol was started. ATT was continued for 18 months, leading to complete clinical recovery.

Case 2

**Figure 2:**
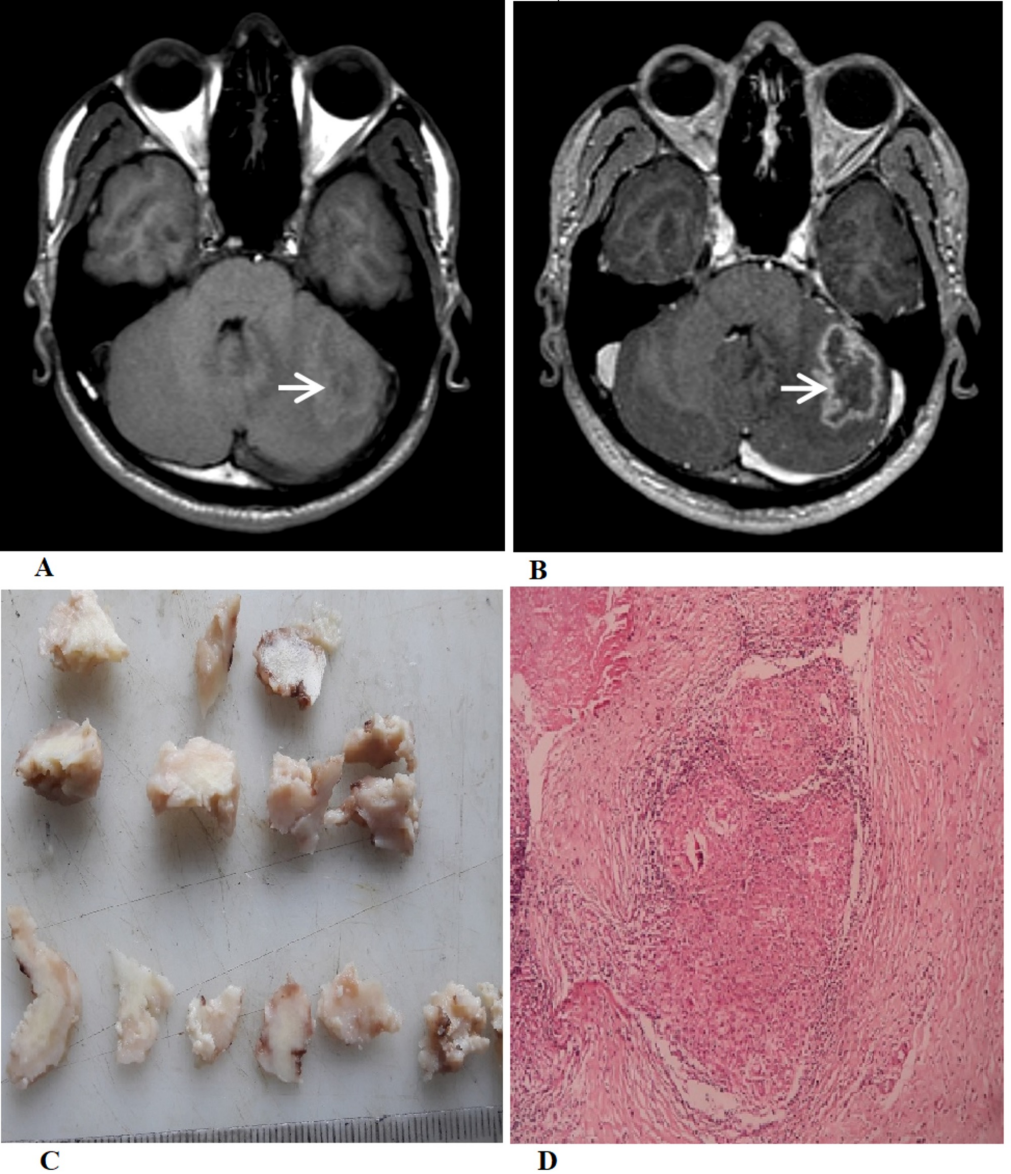
Radiologic and pathologic images (A) Axial T1-weighted non-contrast image shows an iso- to hypointense lesion with a poorly defined peripheral hyperintense rim in the left cerebellar hemisphere (white arrow). (B) Post-contrast T1-weighted axial image shows a peripheral ring enhancement of the lesion (white arrow). (C) The gross specimen shows multiple irregular pieces of grayish-brown tissue, and the cut surface shows grayish-white and cheesy areas. (D) Microscopic examination shows confluent epithelioid cell granulomas bordered by lymphocytes.

A 26-year-old man presented to the hospital with a one-month history of progressive intermittent headache, which was associated with dizziness and vomiting. He also had swaying of the body to the left while walking. There was no history of fever or seizure. The patient was taking ibuprofen but was not on other drugs. On examination, the GCS score was 15. There were cerebellar ataxia and right-sided dysdiadochokinesia. There were no signs of meningism. The patient’s ESR, CRP, and other biochemical tests were normal. A plain chest X-ray was unremarkable. MRI of the brain revealed a peripheral-enhancing solitary space-occupying lesion measuring 3.5 x 3.1 x 2.7 cm^3^ in the left cerebellar hemisphere with significant perilesional edema (Figure 2).

The provisional diagnosis of a posterior fossa tumor was made. Considering the progressive nature of his symptoms with the presence of cerebellar signs, a suboccipital craniotomy and excision of the mass was done. The excised mass was pale with solid consistency. The histopathologic examination found a granulomatous inflammatory lesion consistent with the diagnosis of a tuberculoma. ATT was started and continued for 18 months with complete clinical and radiologic recovery.

## Discussion

Cerebral tuberculoma is a dreaded presentation of tuberculosis that results from hematogenous spread of a distant tuberculous focus. Cerebral tuberculomas may be solitary or multiple and are most commonly seen in the basal parts of the brain [4]. The typical gross features of tuberculomas are small, round, or oval-shaped nodules, ranging from 2 to 12 mm in size [5]. There are only a few published case reports describing giant cerebral tuberculomas in the literature [6-8]. It is extremely rare to find cerebral tuberculomas that are large enough to produce compressive features. Giant cerebral tuberculomas can be easily misdiagnosed as intracranial tumors. Only 30% of patients with cerebral tuberculomas have a suggestive chest radiograph [9]. Furthermore, a cerebrospinal fluid (CSF) analysis may not be contributory, as tuberculosis bacilli are not always observable in the CSF [10]. In both our cases, we could establish the diagnosis of tuberculoma only after a histopathological examination of the excised lesion.

The neuroimaging picture for cerebral tuberculoma is different according to the type of granulomatous lesion. If the tuberculoma comprises granulomas that are non-caseating, the lesion is usually hypointense or isointense to gray matter on T1-weighted images, and hyperintense on T2-weighted MR-images. Caseating granulomas typically have a solid center that is hypointense or isointense on T1-weighted images and isointense on T2-weighted imaging. A central region of T2-hypointensity can be seen because of gliosis and monocyte infiltration. This is a useful finding, as we do not find it in many other intracranial lesions. In post-contrast imaging of tuberculoma, we typically see a peripheral ring-enhancement because of vasogenic edema. Sometimes, central liquefactive necrosis can occur in tuberculoma, which can make differentiation from a cerebral abscess difficult. Some reports have described the ‘target sign,’ which is a ring-enhancing lesion with an additional central area of enhancement or calcification, as characteristic of cerebral tuberculomas [11]. However, this is a nonspecific finding related to central enhancement and may lead to an erroneous diagnosis of cerebral tuberculoma [12]. If available, MR spectroscopy is useful to differentiate tuberculomas from other lesions. In tuberculomas, typical MR spectroscopy findings include a decrease in N-acetylaspartate/creatine and prominent peaks of lipid and lactate [13].

Cerebral tuberculomas should always be considered in the differential diagnosis of solitary intracranial mass lesions. However, the diagnosis is difficult because the neuro-imaging presentation is varied and can be non-specific. In many cases, a definitive diagnosis can only be established with a biopsy of the CNS lesion for histopathology and acid-fast bacilli stain and culture. Antitubercular drugs are usually effective in treating cerebral tuberculomas. A long course of chemotherapy for nine to 18 months is typically required, but total cure rates are high [14]. Surgical intervention may be necessary for situations with acute complications such as obstructive hydrocephalus, large lesions with significant mass effect, brainstem compression, or when the diagnosis is not ensured.

## Conclusions

In regions where tuberculosis is endemic or in patients with a history of prior tuberculosis exposure or infection, cerebral tuberculomas should always be considered in the differential diagnosis of single and multiple space-occupying brain lesions. However, because of its rarity, a giant cerebral tuberculoma can easily be misdiagnosed as an intracranial tumor such as a high-grade glioma. A timely and accurate diagnosis allows the early administration of anti-tubercular drugs, decreases patient morbidity, and can potentially prevent neurosurgical excisions that are not required in all patients.
